# Retraction Note: Chlorantraniliprole (Coragen® 20% SC) exposure induced reproductive toxicity mediated by oxidative stress, apoptosis, and sperm quality deficient in male Wistar rats

**DOI:** 10.1007/s00210-025-04885-3

**Published:** 2025-12-09

**Authors:** Shrouq Adel Mahmoud, Abd El-Wahab El-Ghareeb, Heba Ali Abd El-Rahman

**Affiliations:** Zoology Department, Faculty of Science, Cairo University, Giza, Egypt

**Retraction Note: Naunyn-Schmiedeberg's Archives of Pharmacology (2025) 398:9043-9060**

10.1007/s00210-025-03806-8

The Editor-in-Chief has retracted this article. After publication, concerns have been raised regarding the overlapping elements in the comet assay images of Figure 1. The authors provided raw data files, however, they do not match the corresponding panels. In addition, the data in the tables do not correspond to n=7 as stated in the table legends. The Editor-in-Chief therefore no longer has confidence that the conclusions presented are adequately supported by the data.

Heba Ali Abd El-Rahman does not agree to this retraction. Shrouq Adel Mahmoud and Abd El-Wahab El-Gha have not responded to any correspondence from the editor or publisher about this retraction.

